# Sensitive, homogeneous, and label-free protein-probe assay for antibody aggregation and thermal stability studies

**DOI:** 10.1080/19420862.2021.1955810

**Published:** 2021-08-30

**Authors:** Salla Valtonen, Emmiliisa Vuorinen, Ville Eskonen, Morteza Malakoutikhah, Kari Kopra, Harri Härmä

**Affiliations:** Department of Chemistry, University of Turku, Turku, Finland

**Keywords:** Antibody, aggregation, formulation, homogeneous, label-free, time-resolved luminescence

## Abstract

Protein aggregation is a spontaneous process affected by multiple external and internal properties, such as buffer composition and storage temperature. Aggregation of protein-based drugs can endanger patient safety due, for example, to increased immunogenicity. Aggregation can also inactivate protein drugs and prevent target engagement, and thus regulatory requirements are strict regarding drug stability monitoring during manufacturing and storage. Many of the current technologies for aggregation monitoring are time- and material-consuming and require specific instruments and expertise. These types of assays are not only expensive, but also unsuitable for larger sample panels. Here we report a label-free time-resolved luminescence-based method using an external Eu^3+^-conjugated probe for the simple and fast detection of protein stability and aggregation. We focused on monitoring the properties of IgG, which is a common format for biological drugs. The Protein-Probe assay enables IgG aggregation detection with a simple single-well mix-and-measure assay performed at room temperature. Further information can be obtained in a thermal ramping, where IgG thermal stability is monitored. We showed that with the Protein-Probe, trastuzumab aggregation was detected already after 18 hours of storage at 60°C, 4 to 8 days earlier compared to SYPRO Orange- and UV250-based assays, respectively. The ultra-high sensitivity of less than 0.1% IgG aggregates enables the Protein-Probe to reduce assay time and material consumption compared to existing techniques.

## Introduction

Biologic products are produced from or contain components of living organisms, and they are a rapidly growing class of drugs.^1,2^ There are currently ~100 therapeutic monoclonal antibodies on the market, and over 600 are under clinical studies, anticipating rapid growth in the near future.^3^ The development of biologics is a continuous process where the safety issues must be constantly monitored, even at the stage of final product. In protein-based biologics, aggregation is a major concern. The compromised integrity of the protein drug can be life threatening to patients, as aggregation can lead to increased immunogenicity when the drug is administered.^4,5^ In addition, aggregated protein drugs may lose their activity, and their delivery to the target tissues may be compromised.^6,7^ Thus, it is important to ensure product stability. To control the quality of biologics, stringent regulations have been set by, for example, the U.S. Food and Drug Administration and European Medicines Agency, obligating manufacturers to constantly test the product stability and the level of aggregation.^8,9^ This has led to an increasing need for rapid, easy-to-use, and cost-effective methods to monitor antibody stability and aggregation. In particular, methods suitable for improved high-throughput formulation screening, such as optimization of storage buffers, are urgently needed.

Protein aggregation occurs mainly through interactions of the interior surfaces of the protein. The major mechanisms of protein aggregation are self-association, association due to conformational changes, chemically induced oligomerization, and critical nucleus or surface-induced aggregation. Often these different mechanisms partially unfold proteins, which exposes the buried hydrophobic amino acid residues to the solvent and acts as an intermediate step leading eventually to aggregation. Multiple chemical and physical factors can further induce aggregation, e.g., high concentration, freeze-thaw cycles, temperature, mechanical stress, surface effects, pH, and buffer conditions, such as ionic strength or trace metals.^10,11^ Aggregation is thus a multistep process, which makes it challenging to study.

Protein aggregates are studied with multiple different methods, and aggregate size is the main criterion for choosing the method. Although a single method can provide information on aggregation, multiple methods are required to verify the results and to enable study of aggregates of different sizes and forms.^12^ Chromatography and microscopy are traditional methods for aggregate studies, and both are still widely used. Size exclusion chromatography (SEC) is one of the most applied methods for the detection of protein aggregates. In a SEC column, the size and shape of macromolecules affect their elution through the column. Larger molecules flow past the resin and elute more rapidly, which leads mainly to size-based separation, and makes SEC the most suitable method for relatively small aggregates and multimers.^10,13,14^ Microscopy offers the advantage of visualizing the aggregated sample, giving information about the shape, size, and distribution of aggregates. Electron, atomic force and fluorescence microscopy are methods that potentially enable early aggregate detection.^15–17^ Both SEC and microscopy techniques, however, allow only a limited number of samples to be investigated concurrently, making these methods slow and unsuitable for larger sample panels. These methods also require specific instrumentation and expertise, further limiting wide use.

Biophysical methods are more suitable for these larger sample panels and might even enable high-throughput screening (HTS). Dynamic light scattering (DLS), which determines the hydrodynamic size of aggregates by measuring their diffusion properties in solution, is one of these methods. DLS enables the study of a wide variety of aggregates, but has low sensitivity. It is also unsuitable for quantification, as it gives only information on the particle distribution but not concentration.^10,18,19^ DLS measurements also need to be carefully controlled to avoid contaminants or interference related to, for example, bubble formation. Another scattering-based method widely applied for aggregation monitoring is Rayleigh scattering. In this method, the increase in light scattering at the non-absorbing region is monitored upon protein aggregation. This method is simple and rapid, but, as with DLS, it lacks sensitivity, working in the µM range.^12,20–24^ UV-spectroscopy, on the other hand, uses light scattering by monitoring aggregation based on the turbidity changes caused by the large aggregate particles. Aggregates increase the amount of light scattering compared to samples with native proteins, and thus aggregation is detected as an increase in the absorbance.^25^ These measurements can be performed at, for example, 280 or 350 nm. Commonly, a sample concentration of several mg/mL is required for UV-spectroscopy.^26,27^

Fluorescence-based methods have become increasingly popular due to their simplicity and higher throughput compared to other techniques. ProteoStat, which uses a fluorescent rotor dye, is one of the most widely used assays today. The freely rotating dye is non-fluorescent, but, upon binding to aggregates, the rotational freedom is lost, leading to an increase in the monitored fluorescence.^28,29^ Another fluorescent dye, SYPRO Orange, is also widely applied for protein denaturation and aggregation studies. With intact proteins, SYPRO Orange fluorescence is quenched in an aqueous environment. Protein denaturation exposes the hydrophobic areas of the protein for SYPRO Orange binding, which protects the dye from water and leads to increased fluorescence.^30^ Because aggregation also disrupts the protein structure and makes the hydrophobic core areas accessible to the dye, SYPRO Orange can be applied to monitoring aggregation.^28^ Due to the different mechanisms of interaction with the aggregates (hydrophobicity vs molecular rotor), SYPRO Orange is more sensitive for detecting large protein aggregates, whereas ProteoStat is more suitable for small aggregates, and can even bind to monomeric antibodies.^28,31^ Both introduced methods can be used in a microtiter plate format, enabling HTS assays, although these fluorescent dyes are not specific indicators of antibody aggregation.^28,32,33^ In addition, they still require large amounts of sample, as for example, SYPRO Orange is used for measuring samples with high ng/mL protein concentrations.^34^

The relatively high material consumption is a major problem of all the current HTS-suitable methods.^28,32,33^ The assays are expensive when performed in large quantities, such as in antibody formulation studies, but assay performance can also be affected, as the carry-over antibody storage buffer might affect the results. Aiming to improve on the existing technologies, we developed a sensitive and homogenous method for analyzing antibody aggregates at room temperature (RT). Similar to SYPRO Orange and ProteoStat, this label-free assay requires no conjugation of the target protein, eliminating the effect of labels to the interactions and structural properties. In addition, our Protein-Probe method requires only a fraction of the sample compared to other introduced methods, and it is also suitable for protein stability monitoring, giving additional information when assayed with thermal ramping.^35,36^ Here, we used the Protein-Probe in a proof-of-principle study to monitor a panel of monoclonal antibodies (mAbs) and their heat- or pH-induced aggregation (RT measurements), and also stability in thermal ramping. Both monitored parameters – aggregation behavior and thermal stability – can give important information on therapeutic mAbs. Using several mAbs, we demonstrate the high sensitivity of the Protein-Probe, detecting below 0.1% aggregation from the thermally treated samples. Additionally, we indicate the potential of the Protein-Probe in mAb formulation studies.

## Results

**Figure 1.**
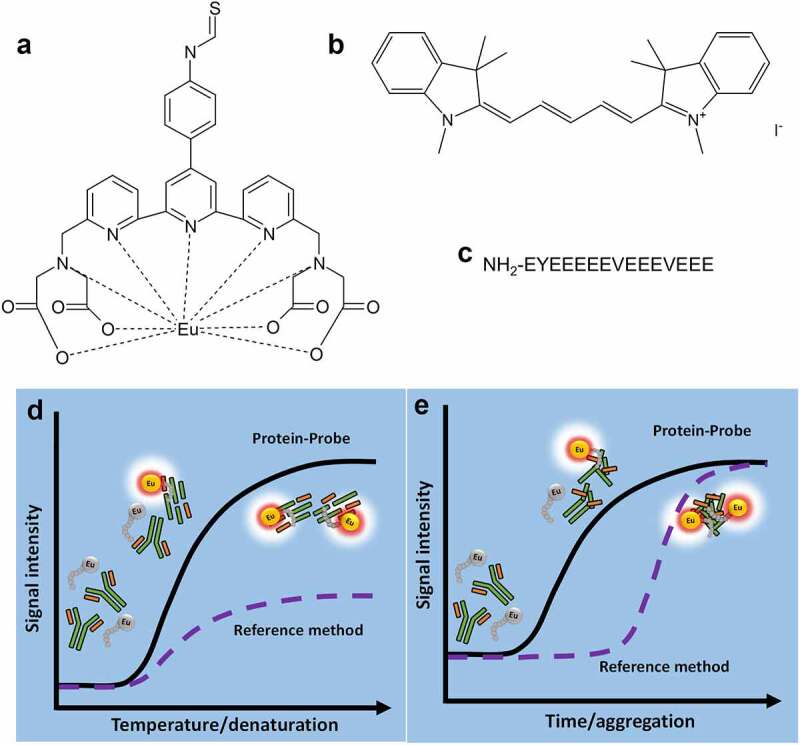
A schematic representation of Protein-Probe assay components and principles for protein stability and aggregation monitoring. The Protein-Probe method has three components: 9-dentate Eu^3+^-chelate ({2,2ʹ,2”,2ʹ“-{[4ʹ-(4ʹ“-isothiocyanatophenyl)-2,2ʹ,6ʹ,2”-terpyridine-6,6”-diyl]bis(methylene-nitrilo)}tetrakis(acetate)}europium(III)) (a), soluble modulator (1,1,3,3,3′,3′-hexamethylindodicarbocyanine iodide, HIDC) (b), and the peptide-probe (EYEEEEEVEEEVEEE) (c). The Eu^3+^-probe is created by labeling the peptide-probe with the Eu^3+^-chelate, and in the Protein-Probe solution, the Eu^3+^-probe is combined with HIDC in pH 4 phosphate-citrate buffer. The Protein-Probe can be used to monitor mAb denaturation by heat (d). When the mAb is in the native form, the Eu^3+^-probe binding is negligible, resulting in a low TRL-signal. Denaturation exposes the hydrophobic inner regions of the mAb, leading to Eu^3+^-probe binding and an increase in the TRL-signal. The melting profile can be monitored by increasing the temperature gradually, enabling melting temperature (T_m_) determination with a significantly lower protein concentration compared to dyes such as SYPRO Orange. The Protein-Probe allows also mAb aggregation monitoring at room temperature (RT) (e). The Eu^3+^-probe does not interact with intact mAbs, but after mAb aggregation, a high Eu^3+^-probe binding induced TRL-signal is monitored. The TRL-signal increase is proportional to the aggregate amount, providing information on the early aggregation process in comparison to dyes such as SYPRO Orange, which detect higher form aggregates

The core of the Protein-Probe method is an Eu^3+^-chelate (Figure 1a) conjugated to the N-terminus of a negatively charged probe-peptide sequence (Figure 1c), i.e., the Eu^3+^-probe. The peptide sequence acts as a sensing element and has minimal interaction with intact proteins at RT. As a protein denatures, for example, upon temperature increase or under the influence of chemical denaturants, its internal hydrophobic patches are exposed, which leads to Eu^3+^-probe interaction and an increase in the time-resolved luminescence (TRL) signal (Figure 1d). The denaturation of the target mAb leads to large protein complex formation via aggregation (Figure 1e), which also promotes Eu^3+^-probe binding and TRL-signal increase at RT.

### The protein-probe method enables aggregation monitoring at room temperature and antibody stability determination at elevated temperatures

To demonstrate the functionality of the Protein-Probe method, we first studied the mAb aggregate-induced effect on the Eu^3+^-probe luminescence lifetime. These properties for the Eu^3+^-probe and Protein-Probe (Eu^3+^-probe solution containing the 1,1,3,3,3′,3′-hexamethylindodicarbocyanine iodide (HIDC)) were monitored in the presence or absence of a thermally aggregated humanized mAb, trastuzumab (Figure 1a-c, Fig. S1). The measured lifetimes for the Eu^3+^-probe alone and in the presence of HIDC (Protein-Probe solution) were 1.2 ± 0.1 and 0.3 ± 0.1 ms, respectively. This results from the Eu^3+^-probe quenching due to HIDC absorption peak overlap with the Eu^3+^-chelate main emission peak at 616 nm (Fig. S2). When the aggregated trastuzumab was measured using the Protein-Probe solution, the lifetime of the Eu^3+^-chelate was prolonged to 0.6 ± 0.1 ms. Even though, due to instrumental sensitivity limitations, the measurements were performed at modified concentrations instead of the ones used in the assays, the increasing trend in luminescence lifetime with the aggregated sample is observed.

**Figure 2.**
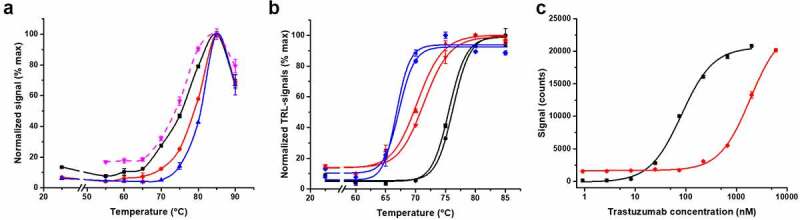
Protein-Probe and SYPRO Orange functionality for mAb denaturation and aggregation monitoring. (a) The melting curves of trastuzumab with the Protein-Probe (solid) and SYPRO Orange (dashed, magenta). Trastuzumab was monitored with the Protein-Probe at 80 (blue), 400 (red), and 2000 nM (black), which showed significant decrease in T_m_ with the increase in mAb concentration. Using 2000 nM trastuzumab, more typical concentration in melting studies, both SYPRO Orange and the Protein-Probe gave highly similar T_m_ values. (b) The melting profiles of three mAbs (80 nM) from two different production batches were investigated with the Protein-Probe. All three mAbs (mAb1, red; mAb2, black; mAb3, blue) showed unique T_m_, inherently characteristic to the mAb, without any major batch-to-batch variation. (c) The sensitivity of the Protein-Probe (black) and SYPRO Orange (red) were compared by using trastuzumab (0.9–6000 nM) as a model mAb. For both assays, trastuzumab was thermally denatured at 85°C before the Protein-Probe and SYPRO Orange addition and the TRL- or luminescence signal monitoring, respectively. The Protein-Probe showed over 100-fold improved sensitivity in comparison to SYPRO Orange. Data represent mean ± SD (n = 3)

At the beginning, thermal denaturation experiments were performed to compare the melting temperatures acquired for trastuzumab (2000 nM) with the Protein-Probe and a reference method, SYPRO Orange. With the Protein-Probe, two other lower concentrations (80 and 400 nM), undetectable with SYPRO Orange, were also measured to monitor if the concentration would affect the T_m_. Significantly, we obtained a 4.7°C higher T_m_ value with 80 nM trastuzumab in comparison to 2000 nM with the Protein-Probe (Figure 2a). However, with 2000 nM trastuzumab, the results obtained using the Protein-Probe and SYPRO Orange were highly similar, with T_m_ values of 76.3 ± 0.6 and 75.1 ± 0.4°C, respectively. At this concentration, the signal-to-background (S/B) values of 13 and 6.0 were obtained using the Protein-Probe and SYPRO Orange, respectively, when the minimum and maximum mAb signals were compared (Figure 2a). Monitoring trastuzumab with the Protein-Probe at a concentration of 80 or 400 nM produced S/B ratios higher than 23, suggesting that the high, micromolar concentration is suboptimal for the Protein-Probe thermal curve measurements.

Based on these results, we selected the 80 nM mAb concentration for further thermal denaturation studies with the Protein-Probe and used three other mAbs to study batch-to-batch variation. These mAbs, which were from two different manufacturing batches, showed no clear batch-dependent changes in T_m_. The S/B ratios, calculated comparing the minimum and the maximum mAb signals, were also not changed (Figure 2b). The average T_m_ values for mAb1, mAb2, and mAb3 were 70.9 ± 0.8, 76.0 ± 0.5, and 66.8 ± 0.4°C, respectively. Interestingly, although there were no batch-to-batch dependent changes in the melting profiles, the signal levels of mAb1, mAb2, and mAb3 varied at RT when measured prior to the denaturation (Figure 2b). This was hypothesized to be due to different levels of mAb aggregation.

After observing the elevated mAb TRL-signal levels at RT, which we assumed were related to aggregation, we determined the assay sensitivity and dynamic range for aggregated mAbs, using SYPRO Orange as a control. These methods were compared side-by-side in a titration using heat-aggregated trastuzumab. It is widely known that heat-denatured mAbs have a tendency to aggregate, and thus we prepared the titration samples by incubating the trastuzumab (0.9–6000 nM) at 85°C for 3 min.^37,38^ The temperature was chosen based on the known high T_m_ of trastuzumab, with the aim of obtaining a fully denatured/aggregated sample. Incubating the trastuzumab sample for a brief period at a high temperature led to similar behavior as aggregating the sample over a long time at a lower temperature (data not shown). After the samples had been aggregated at the elevated temperature, the Protein-Probe or SYPRO Orange were added, followed by signal reading. As calculated from the S/B ratio level of 3 (compared to buffer), the trastuzumab detection limit (LOD) was over 100-fold lower for the Protein-Probe compared to SYPRO Orange (Figure 2c). The calculated LODs for the Protein-Probe and SYPRO Orange were 4.9 and 570 nM, respectively. Based on these results, we selected 2 µM mAb for the future studies with SYPRO Orange, whereas the Protein-Probe was used in a mAb concentrations below 100 nM (Figure 2c).

### The protein-probe is a versatile and sensitive aggregation detection method

To monitor mAb aggregation, we first analyzed 26 different mAbs at RT and in thermal ramping (Table S1). Based on these initial studies, six mAbs with different degrees of aggregation detected at the RT and varying T_m_ values were selected for further studies. We chose one mAb with low T_m_ (60.4 ± 0.3°C) and one with high T_m_ (82.7 ± 0.2°C), whereas the other four mAbs had mid-range T_m_ values between 65 and 71°C.

**Figure 3.**
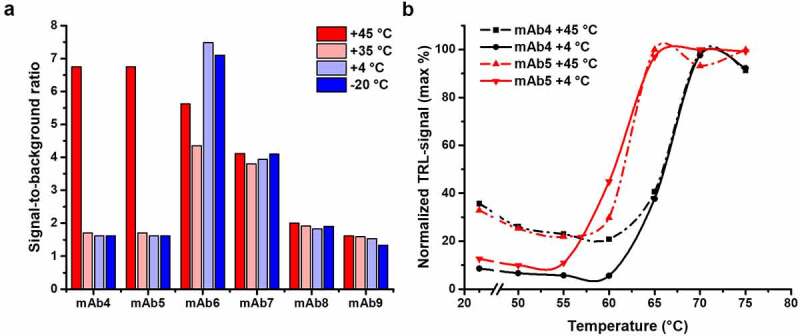
mAb aggregation can be monitored with the Protein-Probe. (a) Six mAbs were stored at different temperatures (−20 to +45°C) to study their tendency to aggregate. mAb4 and mAb5 produced high S/B ratio, when compared to the buffer, after storage at +45°C, indicating a high level of aggregation. mAb6 and mAb 7 had elevated S/B values regardless of the storage conditions. In contrast, mAb8 and mAb9 provided low S/B ratios independent of the storage temperature, suggesting low tendency for aggregation. (b) The thermal stability assays for aggregated and non-aggregated mAbs (80 nM) showed no change in the T_m_. Here, we compared mAb4 (black) and mAb5 (red) stored at +45°C (dashed line) and +4°C (solid line). Storage at +45°C led to high TRL-signal already at low temperatures, compared to +4°C storage. Data represent mean ± SD (n = 2)

The six chosen mAbs (4.8–5.2 mg/mL, 30–34 μM) were all similarly stored for three weeks at four different temperatures (−20, +4, +35, and +45°C) prior to the measurement (Figure 3a). These mAbs were diluted to 80 nM for the aggregation measurements to also enable comparable melting profile determination with the Protein-Probe (Figure 3b, Fig. S3). Of the six mAbs, mAb4–7 showed increased signal at the RT aggregation measurements after being stored at +45°C for three weeks. In addition, mAb6 and mAb7 showed a significant level of aggregation also at the three other storage conditions. We observed no signal changes with mAb8 and mAb9 at any storage temperature, indicating good stability. When the T_m_ values of the thermally stressed mAbs were monitored, no aggregation-related change was observed when compared to the fully soluble protein stored at +4°C (Fig. S3). In Figure 3b, this is highlighted with mAb4 and mAb5, both showing similar levels of aggregation after storage at +45°C. In addition, there was no association between the T_m_ of individual mAbs and their tendency to aggregate at these storage conditions and temperatures below their T_m_ (Fig. S3).

Additionally, to prove that the Protein-Probe can sense the mAb aggregation, mAb4 and mAb10 were analyzed side-by-side with native polyacrylamide gel electrophoresis (PAGE) and the Protein-Probe method (Fig. S4). For this, mAb4 stored either at −20 or +45°C for 3 weeks was selected, as it previously showed a tendency to aggregate (Figure 3). mAb10 stored at +4°C (T_m_ 66.3 ± 1.0°C) was selected as a second mAb, as it showed no aggregation. Additionally, −20°C-stored mAb4 and mAb10 were incubated 3 min at 85°C before the testing to induce aggregation, as was done previously with trastuzumab. As expected, native mAb10 and mAb4 produced a single clear band on the PAGE gel and low signals using the Protein-Probe technique, indicating that they were not aggregated (Fig. S4A). After the thermal denaturation treatment at 85°C, these mAbs were both fully aggregated, which was monitored as the absence of a band on the gel and the accumulation of the dye to the bottom of the loading wells. This is observed because the large aggregates were not able to properly enter the gel. In agreement with these results, the aggregated samples produced on average 32-fold signal increase in the Protein-Probe assay compared to the non-heated samples of the same antibodies (Fig. S4B). The 45°C-stored mAb4 produced a native mAb band, but aggregates were also visible at the edge of the gel. This was in line with the results obtained with the Protein-Probe (Figure 3, Fig. S3, and Fig. S4B), showing increased TRL-signal at RT, but still a visible thermal denaturation curve due to the only partial aggregation. The TRL-signal obtained with the Protein-Probe was between the signals from the fully aggregated and non-aggregated samples, which indicates that the detected melting curves for aggregates samples, e.g., mAb4, are obtained through the non-aggregated population. Thus, we assayed mAb4 thermal denaturation curves using the fully heat-aggregated (3 min at 85°C) mAb4 sample at two concentrations, 20 and 80 nM (Fig. S5). As a control, we used native mAb4 in the same concentrations. While the native mAb produced low TRL-signal at RT and measurable melting curves at both mAb4 concentrations, the opposite was monitored with the heat-aggregated mAb4. Aggregation induced a high TRL-signal at RT and a slight decrease in the observed signal upon heating, producing no thermal denaturation curve (Fig. S5).

**Figure 4.**
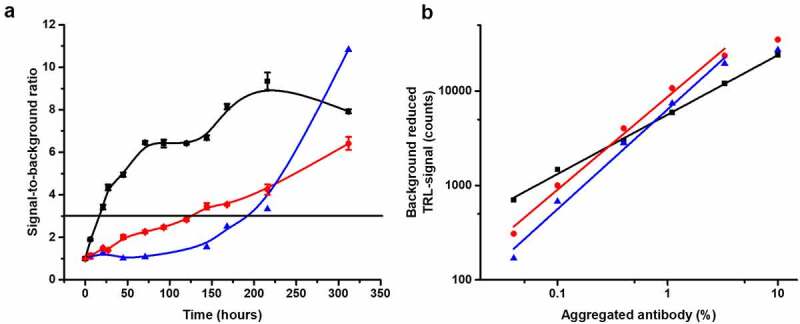
The Protein-Probe enables early and sensitive detection of aggregation. (a) Comparison of the Protein-Probe (black) with SYPRO Orange (red) and UV250 absorbance (blue) for the detection of trastuzumab aggregation. Trastuzumab (5 mg/mL, 32 μM) aggregation was monitored at several time points during a 13-day storage period at 60°C. Detection was performed using 2 µM trastuzumab concentration with all methods, and the earliest time points to detect aggregation (S/B = 3; denoted as a horizontal line) for the Protein-Probe, SYPRO Orange, and UV250 were 18, 123, and 205 hours, respectively. The data is presented as a S/B ratio calculated by comparing the average signals (n = 3) of trastuzumab stored at 60°C or 4°C (non-aggregated control). (b) Linearity and detectability of mAb2 (black), mAb4 (blue) and trastuzumab (red) with the Protein-Probe technique, presented as a function of spiked aggregate percentage. Heat-aggregated mAbs were titrated from 0 to 10% in the presence of monomeric mAb (total concentration 6 µM). The Protein-Probe was capable of monitoring aggregates below 0.1% with all three mAbs. Data is presented as background (non-aggregated mAb) reduced signals, sample n = 3, background n = 6

Next, the Protein-Probe sensitivity for aggregation monitoring was assessed and compared to two reference methods, SYPRO Orange and UV spectroscopy. This UV wavelength of 250 nm was chosen based on our previous observations that using a wavelength shorter than 280 nm improves the detection of aggregates, even though also other types of turbidity measurements have been previously reported.^26,39,40^ Concentrated trastuzumab (5 mg/mL, 32 µM) was stored at 60°C for 13 days and the level of aggregation was monitored multiple times during the storage period at a final 2 µM concentration, which was found suitable for all three methods (data not shown). We chose to perform the assay using accelerated protocol in 60°C due to the high T_m_ of trastuzumab. The earliest time of detection, calculated as S/B = 3 compared to intact non-aggregated trastuzumab (stored at 4°C), was 18 h with the Protein-Probe, 123 h with SYPRO Orange, and 205 h with UV250 (Figure 4a). The analytical LOD for the Protein-Probe, calculated as 3*SD of the blanks (intact trastuzumab), was only 1.2 hours. These results are in line with the previous results showing significantly improved sensitivity with the Protein-Probe compared to SYPRO Orange (Figure 2c).

The Protein-Probe sensitivity for early aggregation events was studied by titrating the amount of aggregated mAbs within intact mAb solutions. This was performed to maintain the total protein concentration and to only change the concentration of the aggregated population (Fig. S4). Trastuzumab, mAb2 and mAb4 were selected for these experiments, as these samples showed low luminescence signal, i.e., low tendency for aggregation at RT (Figure 2b, Figure 4a). To produce 100% aggregated samples, mAb samples were aggregated for 3 min at 85°C, as it was found sufficient based on the PAGE results. Aggregate titration from 0 to 10% was performed by mixing the aggregate with the respective intact mAb and keeping the total mAb concentration at 6 µM. For all mAbs, aggregation below 0.1% was detected with linear range from 0.04 to 3.3% (Figure 4b).

### The protein-probe enables mAb storage buffer formulation studies

**Figure 5.**
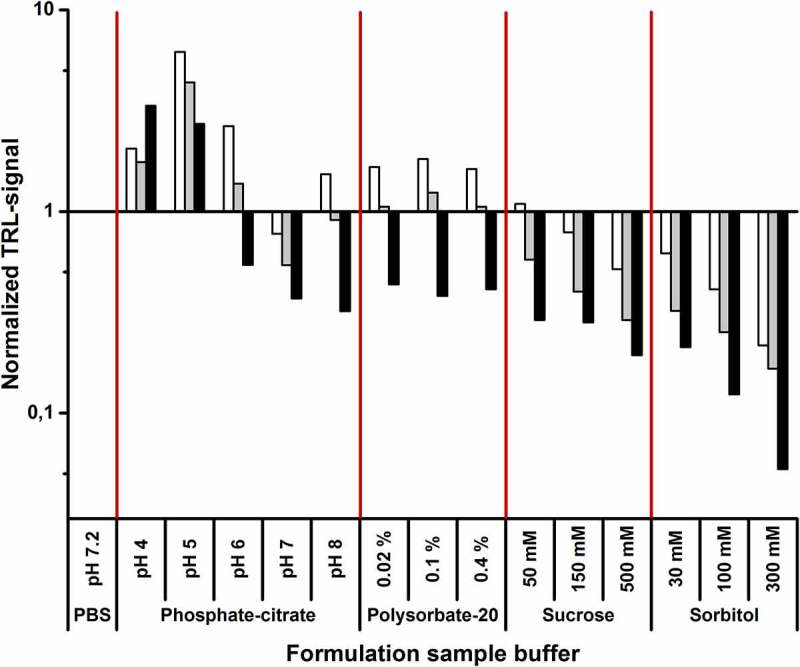
The Protein-Probe as a tool for mAb storage buffer formulation. mAbs were stored in 15 different buffers for four days before monitoring the mAb aggregation level. Trastuzumab (black) was stored at 65°C, and mAb1 (white) and mAb11 (gray) at 45°C. The tested buffers were PBS, phosphate-citrate buffer pH 4–8 with 0.9% (w/v) NaCl, and pH 7 phosphate-citrate buffer with 0.9% (w/v) NaCl supplemented with 0.02–0.4% (v/v) polysorbate-20, 50–500 mM sucrose, or 30–300 mM sorbitol. PBS-normalized values larger than one indicate a high amount of mAb aggregate, and values lower than one indicate an increased mAb stability over the PBS-stored mAb. Both sucrose and sorbitol stabilized all mAbs, and the level of aggregation decreased in a concentration dependent manner. Polysorbate-20, on the other hand, destabilized mAb1 and mAb11, but had a negligible effect on trastuzumab. In addition, mAbs stored in low pH buffers aggregated significantly in comparison to storage buffers with near neutral pH. Data represent PBS-normalized mean values (n = 4)

To simulate the applicability of the method to mAb storage buffer formulation, trastuzumab, mAb1, and mAb11 were stored in 15 different buffers (Table 1) for four days. Trastuzumab, having the highest T_m_ (81.0 ± 0.2°C), was stored at 65°C and the other two mAbs at 45°C to accelerate the aggregation. Thereafter, potential compositional changes were monitored using the Protein-Probe method at RT. Phosphate-buffered saline (PBS; pH 7.2) was chosen as a reference buffer, as it is often used as a storage buffer for mAbs, and the data was normalized against the PBS signal. All other buffers were based on varying phosphate-citrate compositions with pH ranging from 4 to 8, supplemented with 0.9% (w/v) NaCl (154 mM), which is commonly found in mAb storage solutions. A wide pH range was chosen so that aggregation in non-optimal conditions could be monitored with all mAbs with a relatively small number of conditions. For trastuzumab, the degree of aggregation decreased with increasing pH, showing the highest stability at pH 8, whereas in the case of mAb1 and mAb11, the lowest amount of aggregation was observed at pH 7. Surprisingly, mAb1 and mAb11 appeared to be more stable at pH 4 over pH 5, although we expected low pH to promote aggregation also with these mAbs (Figure 5).

**Table 1.** Formulation buffer compositions

Number	Base buffer	pH	Excipient
1	PBS	7.2	NA
2	Citrate-phosphate buffer + 0.9% (w/v) NaCl	4	NA
3	Citrate-phosphate buffer + 0.9% (w/v) NaCl	5	NA
4	Citrate-phosphate buffer + 0.9% (w/v) NaCl	6	NA
5	Citrate-phosphate buffer + 0.9% (w/v) NaCl	7	NA
6	Citrate-phosphate buffer + 0.9% (w/v) NaCl	8	NA
7	Citrate-phosphate buffer + 0.9% (w/v) NaCl	7	0.02% (v/v) polysorbate 20
8	Citrate-phosphate buffer + 0.9% (w/v) NaCl	7	0.1% (v/v) polysorbate 20
9	Citrate-phosphate buffer + 0.9% (w/v) NaCl	7	0.4% (v/v) polysorbate 20
10	Citrate-phosphate buffer + 0.9% (w/v) NaCl	7	50 mM sucrose
11	Citrate-phosphate buffer + 0.9% (w/v) NaCl	7	150 mM sucrose
12	Citrate-phosphate buffer + 0.9% (w/v) NaCl	7	500 mM sucrose
13	Citrate-phosphate buffer + 0.9% (w/v) NaCl	7	30 mM sorbitol
14	Citrate-phosphate buffer + 0.9% (w/v) NaCl	7	100 mM sorbitol
15	Citrate-phosphate buffer + 0.9% (w/v) NaCl	7	300 mM sorbitol

The pH 7 buffer, which showed low signal/aggregation with all three mAbs earlier, was chosen for additive testing. The tested excipients were polysorbate-20 (0.02–0.4% (v/v)) and two sugar-related polyols, sucrose (50–500 mM) and sorbitol (30–300 mM). Under these conditions, polysorbate-20 showed minor destabilization with mAb1 and mAb11, having basically no effect on trastuzumab stability (Figure 5). On the other hand, sucrose and especially sorbitol enhanced the mAb stability compared to the buffer without additive. Supplementing the buffer with 300 mM sorbitol led to the lowest amount of aggregation with all three mAbs (Figure 5).

To further study the effect of pH on aggregation propensity, we monitored an additional six mAbs after storing the samples in phosphate-citrate buffer (pH 4 to 8) containing 0.9% (w/v) NaCl at 45°C for 4 days. We selected IgG_1_ subclass mAb1 and mAb11 as controls, as they previously showed unexpectedly low signal at pH 4, indicating relatively good tolerance of low pH (Figure 5). In addition, we selected two other IgG_1_ mAbs (mAb12 and mAb13), two IgG_2a_ mAbs (mAb14 and mAb15), and two chimeric IgG_1_ mAbs (mAb16 and mAb17) (Fig. S6). Unlike mAb1 and mAb11, these six mAbs had equal or higher aggregation propensity at pH 4 storage buffer compared to pH 5. Generally, the tested mAbs showed the lowest aggregation at pH 6 or pH 7 (Fig. S6).

## Discussion

Antibodies are widely used binding molecules in academy and industry, and their use as protein drugs is ever increasing. Especially in the case of biologics, it is of high importance and legally mandated to closely monitor protein drug quality at several stages, to ensure patient safety and drug/reagent stability. Previously, we demonstrated the Eu^3+^-probe for monitoring protein stability and interactions and protease activity.^35,36,41^ Here, we further developed the Protein-Probe method to detect mAb aggregates.

The Protein-Probe method is based on measuring the TRL-signal of the Eu^3+^-probe, consisting of a protein-binding peptide probe and a conjugated Eu^3+^-chelate (Figure 1a-c). The measurements are performed in a modulation solution containing a quencher molecule, HIDC. The TRL-signal of the free Eu^3+^-probe in the solution is quenched by HIDC, but the probe binding to a denatured/aggregated protein prevents the energy transfer between the Eu^3+^-chelate and HIDC (Fig. S1, Fig. S2). This leads to an increase in the TRL-signal, which provides the basis for mAb aggregation monitoring (Figure 1d-e).

In a typical assay, the sample is first denatured/aggregated independently of the Protein-Probe solution. Then, 4–8 µL of the mAb sample, at a nanomolar concentration, is combined with 65 µL of the modulation solution prior the TRL-signal monitoring. The high volume of modulation solution in comparison to the sample overrides the effects of the sample buffer and, thus, the samples can be prepared into a wide variety of buffer compositions, with different amounts of, for example, detergents and excipients. Because the sample denaturation or aggregation is performed in the relevant sample buffer before the Protein-Probe addition in an end-point fashion, the modulation solution properties do not disrupt the denaturation/aggregation process.

The Protein-Probe had about a 100-fold improved sensitivity for the detection of denatured trastuzumab compared to SYPRO Orange, enabling the use of nanomolar mAb concentrations (Figure 2c). However, we also observed that mAb concentration has an effect on, for example, thermal denaturation data, as shown with the Protein-Probe in the case of trastuzumab, which produced 4.7°C lower T_m_ when assayed in 2000 nM, compared to 80 nM concentration (Figure 2a). This may be explained with higher aggregation tendency, as frequent protein collision at high protein concentration is likely to increase the aggregation of denaturated proteins and, thus, the measured signal. As many of the existing methodologies for protein melting profiling require a high protein concentration, we argue that the data over the years may have reflected down-shifted T_m_ values due to aggregation masking the correct T_m_ values undetectable at high protein concentrations. For 2000 nM trastuzumab, similar T_m_ values were obtained both with Protein-Probe and SYPRO Orange, and the results are in accordance with those reported elsewhere.^42^

When three mAbs from two different production batches were monitored, we observed no batch-dependent change in T_m_, but all mAbs provided characteristic T_m_ values (Figure 2b). However, different TRL-signal levels were observed at RT. Based on our previous findings related to protein-protein interactions, we assumed that these differences could be caused by varying levels of mAb aggregation in the samples.^36^ The Protein-Probe method has a proven high sensitivity in the detection of protein denaturation, and we assumed it can also potentially monitor early aggregation. Our monitoring of six temperature-stressed mAbs at RT with the Protein-Probe indicated that these mAbs had different stability and susceptibility to aggregation (Figure 3a). Two of the mAbs produced aggregation-indicating high TRL-signal only at the highest storage temperature. Additionally, two mAbs aggregated at all temperatures, whereas two mAbs showed no TRL-signal increase indicative of aggregation regardless of storage temperature. This led us to question whether the high TRL-signal at the RT measurement was linked to higher T_m_ and whether aggregation affects the T_m_ of individual mAbs. To our surprise, the partial aggregation did not affect the monitored T_m_ of any of the tested mAbs (Figure 3b, Fig. S3). However, when mAb4 was fully aggregated, the T_m_ value could no longer be obtained, as the aggregated samples yielded saturating Protein-Probe TRL-signal level already at RT, when 80 nM aggregates were used. Signal saturation was not reached with 20 nM mAb4 aggregates at the selected conditions, but in either case, no thermal denaturation curve was observed (Fig. S5). Furthermore, the T_m_ values did not predict mAb aggregation tendency very well (Fig. S3). The observation that some mAbs aggregated at all temperatures, evidently regardless of their characteristic stability, led us to believe that the storage solution used was suboptimal for them.

To confirm that the Protein-Probe measures aggregation, we monitored mAb samples side-by-side on a native PAGE gel and with the Protein-Probe method (Fig. S4). The mAbs had been stored at non-aggregating temperatures (−20 or 4°C), at 45°C, or aggregated using a 3 min incubation at 85°C. The results produced by the two methods were in good agreement: native mAbs produced bands on the gel and low TRL-signals, whereas the incubation at 85°C led to the complete aggregation of the samples and a high TRL-signal. The sample stored for three weeks at 45°C yielded a band on the gel, but aggregates incapable of properly entering the gel were also observed, indicating only partial aggregation. The TRL-signal measured with the Protein-Probe was elevated, but lower than that of the 100% aggregated sample, which demonstrates that the signal levels can be reliably linked to the level of aggregation.

Multiple different methods are often applied in aggregation monitoring, depending on, for example, the expected size of the aggregate, but most of them suffer from high material consumption and suboptimal sensitivity.^12^ To understand the Protein-Probe sensitivity and potential for early monitoring of mAb aggregation, we decided to test it side-by-side with reference methods, UV250 absorbance and SYPRO Orange (Figure 4a).^26,28,32^ As expected, the Protein-Probe detected aggregation considerably earlier than the reference methods, with an analytical LOD (3*SD of intact trastuzumab) of only 1.2 h in these conditions at increased temperature. Thus, we expect that early aggregation detection with the Protein-Probe may shed light on the mAb aggregation process. We expect that not only aggregation, but also the early nucleation and growth phase could potentially be monitored, which would be a substantial improvement over the existing aggregation measurement techniques. Therefore, we next studied the Protein-Probe sensitivity in detecting early aggregation performing a percentual titration with the aggregated mAbs (Figure 4b). The capability of detecting less than 0.1% aggregation indicates that the Protein-Probe is a highly sensitive tool for studying aggregation at an early stage and is the basis for the very low analytical LOD. However, the sensitivity for different types of aggregates is yet to be addressed.

It is important to monitor the stability of injectable biologics, including mAbs, during, for example, drug storage, packaging, and administration. To assess the suitability of the Protein-Probe for buffer formulation, mAbs were stored at elevated temperatures in buffers with pH 4–8 (Figure 5). Detecting mAb aggregation at varying pHs is important because a low pH can be required, such as during downstream processing of mAbs.^43,44^ These tests were first performed with trastuzumab, mAb1, and mAb11, and the lowest level of aggregation was observed near neutral pH for all mAbs. Unexpectedly, mAb1 and mAb11 (both IgG1 subclass) were observed to have better stability at pH 4 compared to pH 5. However, when six additional mAbs of different IgG subclasses were monitored under the same conditions, the aggregation observed at pH 4 was always greater than or equal to the levels at pH 5 (Fig. S6). This suggests that the improved mAb1 stability observed at pH 4 is a specific property of this mAb and also the closely related mAb11, and not a general antibody subclass-dependent trend.

It is known that various chemical compounds, such as salts, sugars, and amino acids, can increase mAb stability, and thus are often used as additives in mAb storage buffers.^45,46^ To briefly study this with Protein-Probe, we selected polysorbate-20 and two sugar-related polyols, sucrose and sorbitol. Polysorbate-20 is a nonionic surfactant, and thus differs from sucrose and sorbitol, but all these additives are commonly used in the formulation of therapeutic mAbs, mainly to prevent protein denaturation and aggregation.^46–48^ Of the tested excipients, sorbitol improved mAb resistance to aggregation the most (Figure 5). The lowest amount of aggregation was achieved when the pH 7 phosphate-citrate buffer was supplemented with 300 mM sorbitol, suggesting that this is an optimal composition for a long-term storage among the tested buffers. Furthermore, these results demonstrate that the Protein-Probe is an efficient method for formulation testing and enables simple and sensitive method applicable for simultaneous testing of high number of samples. At this stage, we did not analyze the aggregate sizes or types in a detailed manner, as the focus was on demonstrating that the Protein-Probe method is capable of monitoring the aggregation of several different mAbs induced by a variety of conditions. Thus, further studies are required to obtain a more thorough understanding of these aspects.

In conclusion, we demonstrated use of the Protein-Probe method for the detection of protein denaturation and aggregation at nanomolar to micromolar concentrations, using sample volumes below 10 µL in a homogeneous microtiter plate format. This method can be used to detect mAb aggregation in minutes at RT, as demonstrated with both murine mAbs and a humanized therapeutic mAb. The Protein-Probe technique can also assess the stability of mAbs, which can be monitored in thermal ramping, enabling the determination of mAb T_m_ values. We found the method to be significantly more sensitive in monitoring early aggregation compared to the used reference methods, SYPRO Orange and UV250. The Protein-Probe was capable of detecting less than 0.1% of aggregate within intact mAb samples, and this increase in sensitivity can potentially enable the monitoring of early nucleation/aggregation events. The storage buffer effect on mAb aggregation was also studied in formulation tests, and different aggregation tendencies were observed depending on the buffer composition and pH. Thus, the Protein-Probe method was demonstrated to be applicable for monitoring both rapid denaturation and slower aggregation over time, as also visualized using PAGE. The Protein-Probe can offer a sensitive, homogenous, and label-free option for simple and ultra-sensitive aggregation monitoring and for formulation testing in a high-throughput compatible manner.

## Materials and methods

A detailed list of materials and instrumentation, Eu^3+^-probe spectral and lifetime characteristics, native PAGE protocol, and formulas used in data analysis are included as Supporting Information (SI). Unless otherwise specified, the assays were performed with Assay Buffer (0.1x PBS + 0.001% Triton X-100) in a volume of 8 µL, with 2–3 replicates. The Protein-Probe solution, containing citrate-phosphate buffer (7.7 mM Na_2_HPO_4_, 6.1 mM citric acid, pH 4) supplemented with 0.01% Triton X-100, 3.5 µM HIDC, and 1 nM Eu^3+^-probe, was added in 65 µL volume. SYPRO Orange was diluted in Assay buffer and added in 2 or 12 µL volume using 1x or 5x final concentration, respectively. The reagents are available upon request.

### Comparison of the protein-probe and SYPRO Orange in thermal denaturation studies

All Protein-Probe experiments were conducted using two-step end-point protocol. The melting curves of mAb1-mAb26 (80 nM), and trastuzumab (80. 400, and 2000 nM) were monitored using the Protein-Probe. The mAb samples were monitored at RT and elevated temperatures by incubating the mAbs for 3 min at 50–95°C using 5°C increments. After the thermal treatment, the Protein-Probe was added, and the TRL-signals were monitored after 5 min incubation at RT. Trastuzumab (2000 nM) was also monitored using SYPRO Orange in a single-step protocol. The samples were combined with SYPRO Orange solution, and the thermal ramping was performed at above. The luminescence signals were measured at each temperature.

The sensitivity of the Protein-Probe was compared to SYPRO Orange by titrating trastuzumab from 0.9 to 6000 nM. Before performing assays with trastuzumab, it was purified with a NAP-5 column, according to the manufacturer’s instructions, and stored at a final concentration of 5 mg/mL in MQ-H_2_O. Samples were incubated for 3 min at 85°C to denature and aggregate trastuzumab, and thereafter the Protein-Probe solution or SYPRO Orange was added for detection. The TRL- or luminescence signals were monitored after 5 min incubation at RT.

### Antibody aggregation monitoring using the protein-probe, SYPRO Orange, and UV250 absorbance methods

From the mAb panel, six mAbs were stored for three weeks at −20°C, +4°C, +35°C, or +45°C in 4.8–5.1 mg/mL (30–34 μM) concentration to induce aggregation and to validate mAb stability. The samples were diluted (80 nM) and monitored at RT and 50–95°C, using 5°C increments. The Protein-Probe was added, and the TRL-signals were monitored after 5 min incubation at RT. Additionally, mAb4 and mAb10 (1 mg/ml, 6 µM) were incubated for 3 min at RT or 85°C, then diluted 1/10 to MQ-H_2_O and analyzed with the Protein-Probe as described above. These samples were also monitored with PAGE, the protocol of which is included in the SI. Samples of −20°C-stored mAb4 (20 and 80 nM) were also aggregated for 3 min at 85°C, and thereafter monitored in thermal ramping as described above.

To compare different methods for mAb aggregation detection, trastuzumab (5 mg/mL, 32 µM) was stored at +60°C for 13 days. During the incubation period, samples were collected at multiple time points and the level of aggregation was monitored using the Protein-Probe, SYPRO Orange, and UV250 absorbance. The samples (2 µM) were incubated with the Protein-Probe or SYPRO Orange, and the TRL- and luminescence signals were measured after 5 min incubation at RT. For UV250, the absorbance at 250 nm was measured using 400 µL volume in a quartz cuvette.

The Protein-Probe sensitivity for aggregation was monitored by titrating aggregated trastuzumab or non-humanized mAb2 or mAb4 with intact mAbs. The mAbs were aggregated by incubating them for 3 minutes at 85°C, which was considered as 100% aggregated samples. The final mAb concentration at all times was kept at 6 µM, and the amount of added mAb aggregate was 0 to 10%. For these tests, the Protein-Probe solution composition was optimized for the higher sample concentration. The Protein-Probe, containing 4 µM HIDC and 1.5 nM Eu^3+^-probe, was added in 65 µL, after which the samples were added in 6 µL. The background samples (0% aggregated) were assayed using six replicates. After brief mixing, the TRL-signals were monitored as previously.

### mAb storage buffer formulation test using the protein-probe method

mAb storage buffer formulation test was performed using the Protein-Probe method, and 15 different buffers (Table 1). Three selected sample mAbs (mAb1, mAb11, and trastuzumab) were stored in 6 µM concentration and incubated at 65°C (trastuzumab) or 45°C (mAb1 and mAb11) for 4 days. In addition, mAb12-mAb17 (6 µM) were tested in formulation buffers 2–6, using similar protocol than with mAb1 and mAb11. All mAbs were also stored at 4°C and used as non-aggregated controls. All samples were 1/10 diluted in MQ-H_2_O for aggregation monitoring, and 4 µL of samples (600 nM) were mixed with 65 µL of Protein-Probe. The plate was briefly mixed, and the TRL-signals were monitored after 5 min, and multiple times during 60 min incubation at RT. All mAbs were assayed using four individual samples.

## Supplementary Material

Supplemental Material

## Abbreviations

DLSDynamic light scattering

HIDC1,1,3,3,3′,3′-hexamethylindodicarbocyanine iodide

HTSHigh-throughput screening

LODLimit of detection

mAbMonoclonal antibody

PAGEPolyacrylamide gel electrophoresis

PBSPhosphate-buffered saline

RTRoom temperature

SDStandard deviation

SECSize-exclusion chromatography

T_m_Melting temperature

TRLTime-resolved luminescence

UVUltraviolet

## Supporting Information

Additional materials and methods and supporting graphs.
