# Supplementary material for: Sensitive, homogeneous, and label-free protein-probe assay for antibody aggregation and thermal stability studies
Source: MAbs. 2021 Aug 30;13(1):1955810. doi: 10.1080/19420862.2021.1955810 (PMC8409793; doi:10.1080/19420862.2021.1955810)
Supplement: Supplemental Material [file KMAB_A_1955810_SM0610.docx]

**Supporting information**

**Sensitive, homogeneous, and label-free protein-probe assay for antibody aggregation and thermal stability studies**

**Salla Valtonen,^a,*^ Emmiliisa Vuorinen,^a^ Ville Eskonen,^a^ Morteza Malakoutikhah,^a^ Kari Kopra,^a^ and Harri Härmä^a^**

1. Department of Chemistry, University of Turku, Vatselankatu 2, 20500 Turku, Finland

*corresponding author email: samaval@utu.fi

**SUPPORTING INFORMATION**

**1. Experimental section**

**1.1 Materials and instrumentation**

**1.2 Eu^3+^-probe lifetime and spectral characterization**

**1.3 Native PAGE for intact and aggregated mAbs**

**1.4 Data analysis**

**2. Supporting results**

**Table S1. Melting temperatures and aggregation level of tested mAbs**

**Figure S1.** **Eu^3+^-probe and Protein-Probe luminescence lifetime with or without trastuzumab**

**Figure S2. Excitation and emission spectras for the Eu^3+^-probe and HIDC modulator**

**Figure S3. Thermal denaturation curves for mAbs stored at different temperatures**

**Figure S4. PAGE and Protein-Probe results for native and aggregated mAbs**

**Figure S5. Thermal denaturation of native and aggregated mAb4**

**Figure S6. mAb stability in storage buffer with different pH**

**3. Supporting references**

**1. Experimental section**

**1.1 Materials and instrumentation**

The 9d Eu^3+^-chelate, {2,2',2",2'"-{[4'-(4'"-isothiocyanatophenyl)-2,2',6',2"-terpyridine-6,6"-diyl]bis(methylene-nitrilo)}tetrakis(acetate)}europium(III) was obtained from QRET Technologies (Turku, Finland) and conjugated according to manufacturer’s instruction to Eu^3+^-probe peptide (NH_2_-EYEEEEEVEEEVEEE) purchased from Pepmic Co., Ltd (Suzhou, China). Purification of the Eu^3+^-probe was performed as described earlier^1^, and concentration was determined using the DELFIA technique and a commercial EuCl_3_ standard from PerkinElmer Life and Analytical Sciences, Wallac (Turku, Finland). NAP-5 columns were purchased from GE Healthcare (Chicago, IL, USA). Trastuzumab was acquired from Roche (Basel, Switzerland). The other antibodies (26 mAbs) used in this study were a kind gift from Oy Medix Biochemica Ab (Espoo, Finland). Absorbance measurements were performed on transparent 96-well plates (TOMTEC Plastics, Budapest, Hungary). Black Framestar 96-well plates, used in thermal ramping assays, were purchased from 4titude (Surrey, U.K.). All other assays were performed in black OptiPlate 384-well microtiter plates (PerkinElmer, Groeningen, Netherlands). Novex WedgeWell 8 % Tris-Glycine pre-cast mini gels were from Invitrogen (Carlsbad, CA, USA) and PageBlue Protein Staining Solution was from Thermo Fisher (Waltham, MA, USA). Other reagents, including analytical-grade solvents, buffer components, SYPRO Orange (5000x), and 1,1,3,3,3′,3′-hexamethylindodicarbocyanine iodide (HIDC) were from Sigma-Aldrich (St. Louis, MO, USA).

Reverse phase liquid chromatography for Eu^3+^-probe purification was performed using Dionex ultimate 3000 LC system (Dionex Corporation, Sunnyvale, CA, USA) and Ascentis RP-amide C18 column (Sigma-Aldrich, Supelco Analytical).^1,2^ Time-resolved luminescence (TRL) emission signals were measured with excitation wavelength of 340 nm, emission wavelength of 620 nm, 400 or 800 µs delay time, and 400 µs integration time. SYPRO Orange luminescence was measured using 485 nm excitation and 590 nm emission wavelengths. These measurements, as well as all spectral characterizations, were performed with Tecan Spark 20M from Tecan Life Sciences (Männedorf, Switzerland). Eu^3+^-probe and Protein-Probe luminescence lifetime measurements were performed using Varian Cary Eclipse Spectrophotometer (Agilent Technologies, Santa Clara, CA, USA). Temperature ramping was performed with a PTC-100 Programmable Thermal Controller (MJ Research, Inc., Watertown, MA). Absorbance measurements were performed in a quartz cuvette using PerkinElmer Lambda 25 (PerkinElmer, Waltham, MA, USA). Gel electrophoresis was run using the XCell SureLock Mini-Cell electrophoresis system (Invitrogen).

**1.2 Eu^3+^-probe lifetime and spectral characterization**

**The excitation and emission spectra were measured for 1 nM Eu^3+^-probe and the Protein-Probe solution, with and without aggregated trastuzumab. Trastuzumab was aggregated at 60 °C for 4 days and used in 110 nM concentration in a final 73 µL volume. The excitation spectrum was monitored from 250 to 500 nm using emission wavelength of 620 nm. The emission scan was performed from 550 to 800 nm with the excitation at 340 nm. Both emission and excitation measurements were performed using 400 µs delay and integration times. In addition, the excitation spectrum of 3.5 µM HIDC was monitored from 450 to 680 nm using emission wavelength of 700 nm. HIDC emission spectrum was monitored from 630 to 850 nm using excitation at 618 nm. HIDC absorption was measured from 400 to 750 nm. All spectra were measured using 5 nm bandwidth for every 1 nm.**

**The lifetimes of 50 nM Eu^3+^-probe, the Protein-Probe (50 nM Eu^3+^-probe + 2 µM HIDC), and the Protein-Probe in the presence of 1 µM aggregated trastuzumab were monitored using 40 µL sample volume in a quartz cuvette. Excitation and emission wavelengths were 340 and 615 nm, respectively. Lifetime was monitored using 0.1 ms delay and gate times, and the data was collected from 0.1 to 3 ms.**

**1.3 Native PAGE for intact and aggregated mAbs**

Native PAGE was run on 8 % Tris-Glycine pre-cast mini gels in a buffer containing 25 mM Tris base and 192 mM glycine, pH 8.3. mAb samples (1 mg/ml) were prepared into MQ-H_2_O and some of the samples were further incubated 3 min at 85 °C before they were combined 1:1 with sample buffer (100 mM Tris-HCl, 10 % glycerol, 0.0025 % bromophenol blue, pH 8.5). Proteins were loaded onto the gel in 10 µl, 5 µg/lane, and the electrophoresis was performed for 2.5 h with a constant voltage of 200 V. The gel staining was performed with PageBlue Protein staining solution according to the manufacturer’s instructions.

**1.4 Data analysis**

The S/B ratio was calculated as µ_max_/µ_min_ and coefficient variation (CV%) as (σ/µ) x 100. In these formulas, µ is the mean value and σ is the standard deviation (SD). The denaturation temperatures and sensitivities were calculated based on the data fitted using standard sigmoidal and linear fitting functions. The data were analyzed using Origin 2016 (OriginLab, Northampton, MA).

**2. Supporting results**

**Table S1. Melting temperatures and aggregation level of tested mAbs**

Table S1. The types, subclasses, obtained melting temperatures, and aggregation status of all the mAbs monitored with the Protein-Probe. The T_m_ values ranged from 60.4 to 82.7 °C, and two of the mAbs were judged to be slightly aggregated (estimated aggregation <1 %), as an elevated TRL-signal was measured at RT.

| mAb | mAb type | mAb subclass | Tm °C | Aggregation in +4 °C storage |
| --- | --- | --- | --- | --- |
| mAb1 | Murine | IgG_1_ | 70.9 ± 0.8 | - |
| mAb2 | Murine | IgG_1_ | 76.0 ± 0.5 | - |
| mAb3 | Murine | IgG_1_ | 66.8 ± 0.4 | - |
| mAb4 | Murine | IgG_1_ | 65.4 ± 0.1 | - |
| mAb5 | Murine | IgG_1_ | 60.4 ± 0.3 | - |
| mAb6 | Murine | IgG_1_ | 68.8 ± 0.4 | + |
| mAb7 | Murine | IgG_1_ | 65.7 ± 0.1 | + |
| mAb8 | Murine | IgG_1_ | 70.6 ± 0.5 | - |
| mAb9 | Murine | IgG_1_ | 82.7 ± 0.2 | - |
| mAb10 | Murine | IgG_1_ | 66.3 ± 1.0 |  |
| mAb11 | Murine | IgG_1_ | 75.3 ± 0.2 | - |
| mAb12 | Murine | IgG_1_ | 61.5 ± 0.7 | - |
| mAb13 | Murine | IgG_1_ | 68.3 ± 0.4 | - |
| mAb14 | Murine | IgG_2a_ | 73.0 ± 0.7 | - |
| mAb15 | Murine | IgG_2a_ | 75.5 ± 0.3 | - |
| mAb16 | Chimeric | IgG_1_ | 60.9 ± 0.4 | - |
| mAb17 | Chimeric | IgG_1_ | 70.0 ± 0.8 | - |
| mAb18 | Murine | IgG_1_ | 67.6 ± 0.7 | - |
| mAb19 | Murine | IgG_2a_ | 74.2 ± 0.6 | - |
| mAb20 | Murine | IgG_1_ | 72.0 ± 0.9 | - |
| mAb21 | Murine | IgG_1_ | 78.0 ± 0.7 | - |
| mAb22 | Murine | IgG_1_ | 71.5 ± 0.4 | - |
| mAb23 | Murine | IgG_1_ | 77.0 ± 0.5 | - |
| mAb24 | Murine | IgG_1_ | 80.0 ± 0.6 | - |
| mAb25 | Murine | IgG_1_ | 60.4 ± 1.0 | - |
| mAb26 | Murine | IgG_1_ | 74.6 ± 1.1 | - |
| Trastuzumab | Humanized | IgG_1_ | 81.0 ± 0.2 | - |
| - = no aggregation, + = minor aggregation, ++ = moderate aggregation, +++ = major aggregation | | | | |

**Figure S1. Eu^3+^-probe and Protein-Probe luminescence lifetime with and without trastuzumab**

**Figure S1.** Lifetime of the Eu^3+^-probe and the Protein-Probe with and without trastuzumab. The lifetimes of 50 nM Eu^3+^-probe (black), the Protein-Probe solution (50 nM Eu^3+^-probe + 2 µM HIDC; red), and the Protein-Probe solution with 1 µM of aggregated trastuzumab (blue) were monitored. The Eu^3+^-probe lifetime was considerably reduced when HIDC was added. However, when the lifetime of the Protein-Probe solution was monitored in the presence of trastuzumab, it was increased due to the Eu^3+^-probe being protected from the quenching effect of the HIDC. The lifetime for Eu^3+^-probe, 1.2 ± 0.1 ms, was found to be in the expected range.^3^ Due to instrument limitations, lifetimes measured with the Protein-Probe are obtained using high reagent concentrations and atypical reagent ratios, and are thus not directly comparable to the assay data, only indicative.

**Figure S2. Excitation and emission spectra for the Eu^3+^-probe and HIDC modulator**

**Figure S2.** Luminescence excitation and emission spectra of the Eu^3+^-probe and HIDC, and absorbance spectrum of HIDC. The emission of 1 nM Eu^3+^-probe (black), and HIDC (3.5 µM) excitation (red), emission (blue), and absorbance (magenta, dashed line) spectra were monitored from 450 to 850 nm. The Eu^3+^-probe emission maximum was at 616 nm, and the HIDC excitation and emission maxima were found in 635 nm and 656 nm, respectively. The HIDC absorption maximum was 636 nm. The Eu^3+^-probe emission spectrum overlaps with the HIDC excitation and absorption spectra, enabling an efficient quenching of the unbound probe.

**Figure S3. Thermal denaturation curves for mAbs stored at different temperatures**

**Figure S3.** Melting curves of six mAbs stored at four different temperatures. mAb4–9 were stored in 4.8–5 mg/ml (30–34 μM) concentration at different temperatures (-20 °C (blue), +4 °C (black), +35 °C (grey), or +45 °C (red)) for three weeks. Melting curves were measured for individual mAbs at 80 nM concentration in 8 µL volume. The diluted mAb samples were monitored at RT or incubated for 3 min at 50-95 °C using 5 °C increments, before adding 65 µL of the Protein-Probe solution. The TRL-signals were monitored after 5 min incubation at RT. The storage temperature did not affect the T_m_ values, but a significant change was observed in the signals monitored at RT before the heating cycle. This indicates varying level of aggregation. Notably, the TRL-signal measured at RT was considerably elevated for mAb4 (A) and mAb5 (B) when stored at +45 °C, but not due to the other storage temperatures. This indicates that mAb4 and mAb5 are only susceptible to aggregation when stored at elevated temperatures. mAb6 (C) and mAb7 (D) showed elevated signals regardless of the storage temperatures, suggesting that these mAbs were already aggregated during the production or early storage. Low TRL-signal was measured for mAb8 (E) and mAb9 (F) at all temperatures, exhibiting resilience to aggregation. Data represent mean ± SD (n=2).

**Figure S4. PAGE and Protein-Probe results for native and aggregated mAbs**


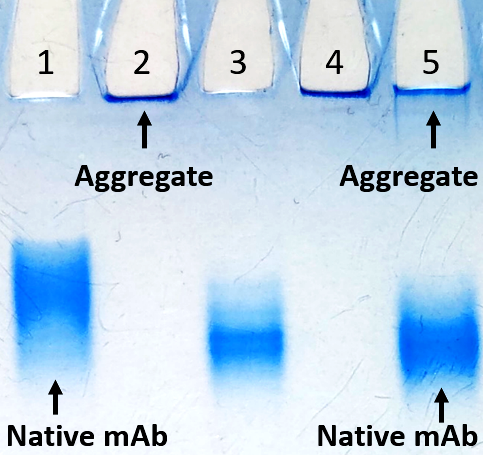


**A**

**Figure S4.** Comparison of native PAGE and the Protein-Probe detection in aggregate characterization. A) 5 µg of differently treated mAb4 and mAb10 were loaded to a 8 % tris-glycine gel, which was run for 2.5 h with a 200 V constant current. The analyzed samples were 4 °C-stored mAb10 without (lane 1) or with (lane 2) heat aggregation (3 min at 85 °C) prior to loading, -20 °C stored (3 weeks) mAb4 without (lane 3) or with (lane 4) heat aggregation, and 45 °C-stored (3 weeks) mAb4 without further threatment (lane 5). Clear bands were observed on lanes 1, 3, and 5, indicating that the mAbs were in their monomeric form. Additionally, aggregated mAb4, which could not enter the gel, was observed at the upper edge of lane 5. This indicates that the 3-week storage of mAb4 at 45 °C induced partial aggregation, as seen also in Fig. 3A and Fig. S3A. The brief incubation at 85 °C produced fully aggregated mAb, observed as a band at the top edge of the gel on lanes 2 (mAb10) and 4 (mAb4). B) The same samples were analyzed with the Protein-Probe using 600 nM samples, and the results were in good agreement with the PAGE results. Well stored mAb10 (4 °C storage) and mAb4 (-20 °C storage) produced low signal, whereas 3 min incubation at 85 °C led to, on average, a 32-fold increase in the observed TRL-signal level compared to the non-aggregated samples. The 45 °C-stored mAb4 produced 12-fold signal compared to the -20 °C-stored sample, indicating partial aggregation, in agreement with the PAGE results. Data represent mean ± SD (n=3).

**Figure S5. Thermal denaturation of native and aggregated mAb4**

**Figure S5.** The thermal curves of native and aggregated mAb4 monitored with the Protein-Probe. Non-aggregated mAb4 (stored at -20 °C) was used as a sample at two concentrations, 20 nM (red) and 80 nM (black), to visualize aggregate function upon heating. Thermal denaturation experiments were performed using a native mAb4 (dashed line) or fully heat aggregated (3 min at 85 °C) samples (solid line). Samples were monitored with the Protein-Probe at RT and at elevated temperatures between 50 and 75 °C, with 5 °C measurement interval. The native mAb produced clear thermal curves at both sample concentrations, giving the expected T_m_ values of 66.4 ± 1.0 °C and 66.3 ± 0.1 °C for 20 and 80 nM mAb4, respectively. The heat-aggregated sample produced maximal TRL-signal already at RT, and the signal level decreased slightly at increasing temperatures, finally reaching the peak signal of the thermal curve of the non-preaggregated samples. Thus, no mAb melting curve could be observed for the fully aggregated samples, only for the sample with no or partial aggregation, as seen in Figure S3A. Data represent mean ± SD (n=3).

**Figure S6. mAb stability in storage buffer with different pH**

**Figure S6.** Effect of storage buffer pH on mAb aggregation using variety of different types of mAbs. After mAb1 and mAb11 produced unexpectedly low TRL-signal after storage at buffer with pH 4, the storage buffer pH was studied with two other IgG_1_ mAbs, two IgG_2a_ mAbs, and two chimeric mAbs. These mAbs were stored at +45 °C for 4 days in phosphate-citrate buffer with pH 4–8, supplemented with 0.9% NaCl, and the aggregation was then monitored using the Protein-Probe. Storage in pH 4 buffer resulted in the highest mAb aggregation level, except for mAb16, which showed nearly equal aggregation level at pH 4 and 5. Thus, it appears that the low aggregation tendency of mAb1 and mAb11 at pH 4 compared to pH 5 was not IgG-class-dependent, but this instead was a property of these two mAbs. Storage in buffers with pH 6-8 resulted in a nearly equal low level of aggregation, although mAb1 and mAb16 showed some aggregation at pH 6 and pH 8, respectively. Data represent mean ± SD (n=4).

**3. References**

1. Vuorinen E, Valtonen S, Eskonen V, Kariniemi T, Jakovleva J, Kopra K, Härmä H. Sensitive Label-Free Thermal Stability Assay for Protein Denaturation and Protein-Ligand Interaction Studies. Anal Chem 2020; 92:3512–6.

2. Valtonen S, Vuorinen E, Kariniemi T, Eskonen V, Le Quesne J, Bushell M, Härmä H, Kopra K. Nanomolar Protein–Protein Interaction Monitoring with a Label-Free Protein-Probe Technique. Anal Chem 2020; 92:15781–8.

3. Latva M, Takatalo H, Mukkala V-M, Matachescu C, Rodriguez-Ubis JC, Kankare J. Correlation between the lowest triplet state energy level of the ligand and lanthanide(III) luminescence quantum yield. J Lumin 1997; 75:149–69.
